# Stability-Driven Osteoporosis Screening: Multi-View Consensus Feature Selection with External Validation and Sensitivity Analysis

**DOI:** 10.3390/jcm15020677

**Published:** 2026-01-14

**Authors:** Waragunt Waratamrongpatai, Watcharaporn Cholamjiak, Nontawat Eiamniran, Phatcharapon Udomluck

**Affiliations:** 1 School of Medicine, University of Phayao, Phayao 56000, Thailand; waragunt@hotmail.com; 2 School of Science, University of Phayao, Phayao 56000, Thailand; watcharaporn.ch@up.ac.th (W.C.); nontawatskip@gmail.com (N.E.)

**Keywords:** osteoporosis screening, feature selection, machine learning, external validation, risk prediction models

## Abstract

**Background/Objectives:** Osteoporosis is a major global health concern, and early risk assessment plays a crucial role in fracture prevention. Although demographic, clinical, and lifestyle factors are commonly incorporated into screening tools, their relative importance within data-driven prediction frameworks can vary substantially across datasets. Rather than aiming to identify novel predictors, this study evaluates the stability and behavior of established osteoporosis risk factors using statistical inference and machine learning-based feature selection methods across heterogeneous data sources. We further examine whether simplified and near-minimal models can achieve predictive performances comparable to that of full-feature configurations. **Methods:** An open-access Kaggle dataset (*n* = 1958) and a retrospective clinical dataset from the University of Phayao Hospital (*n* = 176) were analyzed. Feature relevance was assessed using logistic regression, likelihood ratio testing, MRMR, ReliefF, and unified importance scoring. Multiple predictor configurations, ranging from full-feature to minimal and near-minimal models, were evaluated using decision tree, support vector machine, k-nearest neighbor, naïve Bayes, and efficient linear classifiers. External validation was performed using hospital-based records. **Results:** Across all analyses, age consistently emerged as the dominant predictor, followed by corticosteroid use, while other variables showed limited incremental predictive contributions. Simplified models based on age alone or age combined with medication-related variables achieved performances comparable to full-feature models (accuracy ≈91% and AUC ≈ 0.95). In addition, near-minimal models incorporating gender alongside age and medications demonstrated a favorable balance between discrimination and computational efficiency under external validation. Although overall performance declined under distributional shift, naïve Bayes and efficient linear classifiers showed the most stable external behavior (AUC = 0.728–0.787). **Conclusions:** These findings indicate that stability-driven feature selection primarily reproduces well-established epidemiological risk patterns rather than identifying novel predictors. Minimal and near-minimal models—including those incorporating gender—retain acceptable performances under external validation and are methodologically efficient. Given the limited size and single-center nature of the external cohort, the results should be interpreted as preliminary methodological evidence rather than definitive support for clinical screening deployment. Further multi-center studies are required to assess generalizability and clinical relevance.

## 1. Introduction

Osteoporosis is a chronic, progressive skeletal disorder characterized by decreased bone mass and microarchitectural deterioration of bone tissue, resulting in increased bone fragility and fracture risk [1]. According to the World Health Organization (WHO), osteoporosis is considered a major global health problem, affecting more than 200 million people worldwide and contributing to approximately 8.9 million fractures annually, including hip, vertebral, and wrist fractures that account for substantial morbidity, mortality, and healthcare expenditures [2,3,4]. Its prevalence rises sharply with age, particularly among postmenopausal women due to estrogen deficiency and accelerated bone turnover [5,6]. Hip fractures, in particular, carry a one-year mortality rate approaching 20–30%, creating a substantial clinical and socioeconomic burden globally [7,8].

Early identification of individuals at risk is critical for timely preventive interventions and fracture reduction. Dual-energy X-ray absorptiometry (DXA) remains the gold standard for diagnosing osteoporosis and measuring bone mineral density (BMD) [9]; however, it is limited by cost, radiation exposure, lack of availability in rural or resource-limited regions, and reduced accessibility in primary care settings [10,11,12]. Additionally, reliance on BMD alone does not fully account for the multifactorial nature of osteoporosis, which is influenced by lifestyle behaviors, hormonal status, genetic predisposition, comorbidities, and long-term medication use—such as glucocorticoids—that significantly alter bone metabolism [13,14,15,16].

To address these challenges, clinical risk assessment tools such as Fracture Risk Assessment Tool (FRAX) have been developed to estimate fracture probability using demographic and clinical factors [17,18]. While widely adopted, these tools may suffer from population-specific calibration issues and limited adaptability to heterogeneous clinical environments [19,20]. Consequently, there is growing interest in artificial intelligence (AI) and machine learning (ML) approaches capable of integrating high-dimensional, multi-source data to enhance osteoporosis screening, fracture risk prediction, and clinical decision-making [21,22,23,24].

AI-driven models have shown promise in analyzing DXA images, CT scans, X-rays, electronic medical records (EMR), and lifestyle datasets to predict osteoporosis and fragility fractures with improved accuracy and scalability [25,26,27,28]. Deep learning systems can extract subtle bone quality features beyond BMD, whereas traditional ML algorithms provide interpretable risk factor assessment conducive to clinical adoption [29,30,31]. However, a major limitation in current AI-based osteoporosis research is the lack of reproducibility across diverse datasets, susceptibility to model overfitting, and inadequate external validation—issues that restrict their translation into real-world fracture prevention pathways [32,33,34].

Feature selection plays a central role in developing reliable prediction models. Recent advances highlight the importance of stability-driven feature selection, where predictors are evaluated across repeated subsampling, bootstrapping, or resampling procedures to ensure robustness against data perturbations and multicollinearity [35,36]. Multi-view learning—where predictors are grouped into clinically meaningful domains such as demographics, lifestyle, laboratory biomarkers, and medication history—offers additional advantages by preserving complementary information across heterogeneous data sources [37,38]. Incorporating multi-view consensus mechanisms further enhances model interpretability, reduces the influence of noisy or redundant features, and strengthens generalizability across heterogeneous patient cohorts, as demonstrated in recent machine learning-based medical decision support studies [39,40,41,42]. The present study integrates two complementary datasets to evaluate these methodological innovations. The first dataset is an open-access resource from Kaggle entitled “Lifestyle Factors Influencing Osteoporosis”, comprising 1958 records with demographic, lifestyle, and clinical risk variables [43]. This dataset is not intended to represent population-level epidemiology but is used exclusively as a methodological benchmark to examine feature stability behavior across different machine learning models. The second dataset consists of retrospective clinical records from the University of Phayao Hospital (176 patients; data collected between 2018 and 2025), incorporating clinically relevant fracture-related risk factors such as age, prior smoking exposure, and corticosteroid (prednisolone) use, and serves as the primary source for clinically meaningful interpretation. The combination of open-access population-based data and real-world EMR data provides a unique opportunity to assess model robustness, cross-population reproducibility, and sensitivity to distributional shifts—key requirements for deploying AI tools within fracture management pathways [44,45,46].

This study proposes a stability-driven, multi-view consensus feature selection framework designed to examine how different statistical and machine learning-based feature selection strategies behave across heterogeneous datasets, rather than to identify novel clinical predictors of osteoporosis. The framework is coupled with systematic factor-removal experiments and subgroup-based sensitivity analyses to assess feature robustness, stability, and interpretability under varying data conditions. External validation using hospital electronic medical record (EMR) data enables an unbiased evaluation of model performance in real-world clinical settings, addressing generalizability concerns frequently reported in prior machine learning studies. The expected contribution of this work lies in strengthening methodological rigor, improving transparency in feature selection behavior, and supporting the development of parsimonious and interpretable screening models suitable for community-based applications. Ultimately, this approach provides a methodological foundation for reliable fracture risk stratification while emphasizing cautious interpretation of dominant predictors arising from dataset characteristics rather than independent biological discovery.

## 2. Materials and Methods

### 2.1. Study Design

This study employed a cross-sectional, retrospective, multi-dataset analytical design integrating open-access data and real-world clinical records. The primary objective was not to discover novel clinical predictors of osteoporosis, but to systematically evaluate the stability, robustness, and behavior of different statistical and machine learning-based feature selection strategies across heterogeneous datasets. To this end, a stability-driven, multi-view consensus feature selection framework was implemented, complemented by factor removal experiments, subgroup-based sensitivity analyses, and external validation. This design follows current methodological recommendations emphasizing reproducible machine learning pipelines and independent validation to assess generalizability, mitigate dataset-driven artifacts, and reduce overfitting in clinical prediction models [44].

### 2.2. Data Sources and Descriptives

#### 2.2.1. Data Sources

(a) Open-Access Dataset (Kaggle)

The primary dataset was obtained from the publicly accessible Kaggle repository “Lifestyle Factors Influencing Osteoporosis”, comprising 1958 participants. This dataset contains demographic, lifestyle, nutritional, hormonal, and medical variables related to osteoporosis risk and was used exclusively as a methodological testbed to evaluate feature selection stability and model behavior across machine learning algorithms, rather than for population-level inference. All records were complete, with no missing values. Source: https://www.kaggle.com/datasets/amitvkulkarni/lifestyle-factors-influencing-osteoporosis (accessed on 20 August 2025).

(b) Retrospective Clinical Dataset (Hospital-Based)

An independent external validation dataset was derived from retrospective clinical records at the University of Phayao Hospital collected between 2018 and 2025. A total of 176 patients were included, with variables capturing age, sex, smoking history, corticosteroid/prednisolone use, comorbidities, and confirmed osteoporosis diagnosis based on BMD or clinician assessment.

This combination of datasets allows evaluation of model robustness between population-level and real-world clinical settings.

#### 2.2.2. Descriptive Statistics

Descriptive characteristics of key variables—particularly age, categorical risk factors, and osteoporosis distribution—were summarized using Jamovi (Version 2.7.9.0). Statistical outputs are derived directly from the analysis report as the following baseline characteristics Table 1.

Table 1 shows baseline characteristics of the 1958 participants from the Kaggle osteoporosis dataset, comparing non-osteoporosis and osteoporosis groups. Age showed a marked difference between groups, whereas most categorical variables displayed balanced distributions across both groups.

To further explore the distributional differences in age between individuals with and without osteoporosis, additional graphical analyses were performed. These visualizations provide an intuitive overview of how age is distributed across outcome groups and evaluate whether the assumption of normality is appropriate for downstream statistical modeling.

**Figure 1:**
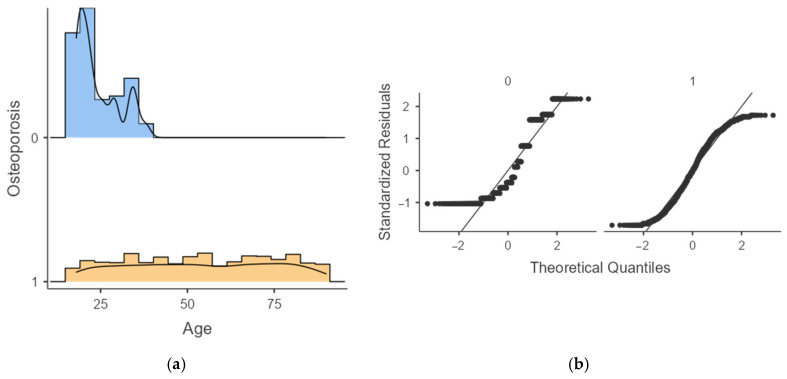
Age distribution (**a**) and Q–Q plots (**b**) for osteoporosis vs. non-osteoporosis groups. The non-osteoporosis group shows a younger and narrower age range, while the osteoporosis group displays an older and wider distribution, with both groups deviating from normality.

From Figure 1, the graphical analysis highlights clear differences in age patterns between groups. Individuals without osteoporosis displayed a narrowly clustered age distribution centered around early adulthood, whereas those with osteoporosis exhibited a substantially wider and older age range, extending into late adulthood. Q–Q plots further revealed pronounced deviations from normality in both groups, suggesting that age is not normally distributed and should be analyzed using non-parametric or model-based approaches rather than parametric methods relying on normality assumptions. These findings reinforce age as a leading discriminative factor for osteoporosis status within the dataset.

### 2.3. Model Development

Model development followed a stability-driven framework using multiple predictor configurations derived from statistical tests and machine learning feature selection. Nine models (Cases 1–9), ranging from full-feature to ultra-minimal sets, were constructed to evaluate the effect of progressive feature reduction.

Five machine learning algorithms were trained for each case—Decision Tree, Support Vector Machine (SVM), k-Nearest Neighbors (KNN), Naïve Bayes, and Efficient Linear—representing diverse learning paradigms. All models were developed and internally validated using the Kaggle dataset (*n* = 1958) with stratified hold-out evaluation. Performance metrics included accuracy, precision, recall, F1-score, and Area Under the Curve (AUC).

Special emphasis was placed on simplified models, particularly those using Age alone or Age + Medications, to determine whether minimal predictor sets could match the performance of more complex models. Models demonstrating high internal stability and strong discriminative performance were selected for subsequent external validation using the hospital dataset.

### 2.4. Workflow

**Figure 2:**
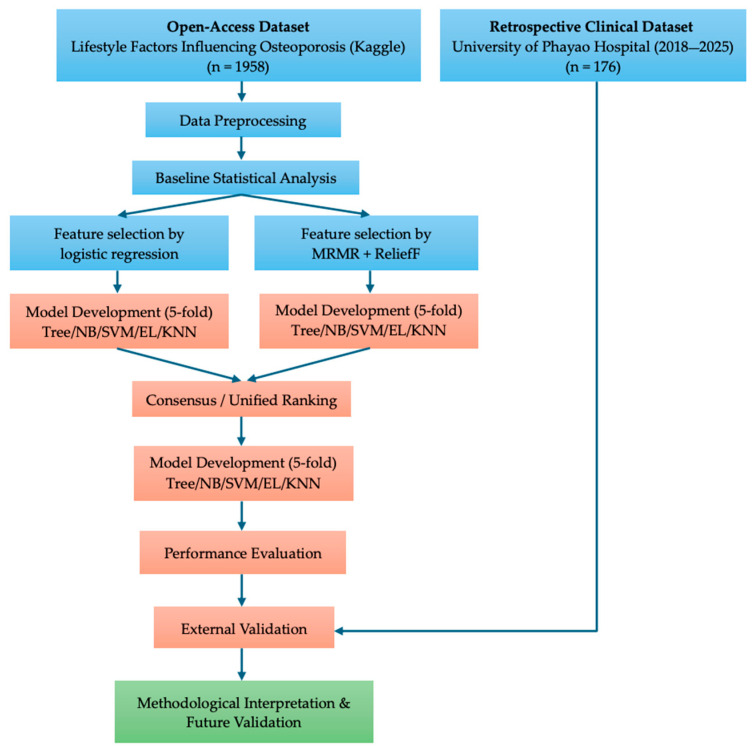
Overall workflow of the stability-driven osteoporosis prediction framework.

Figure 2 illustrates the complete analytical pipeline employed in the study, beginning with data acquisition from open-access and retrospective clinical sources, followed by data preprocessing and baseline statistical analysis. Feature selection was conducted using complementary statistical and machine learning approaches, with a unified consensus ranking guiding model development across multiple predictor configurations and classification algorithms. Model performance was assessed through internal evaluation and preliminary external validation. The final stage emphasizes methodological interpretation and highlights directions for future clinical validation rather than direct deployment.

## 3. Results

### 3.1. Statistical Analysis of Predictors

From Table 2, analysis of all predictors revealed that age is overwhelmingly the strongest determinant of osteoporosis, with an exceptionally high likelihood ratio (χ^2^ = 1329.6 and *p* < 0.001) and a highly significant logistic regression coefficient (OR = 1.17 per year). This indicates that each additional year of age increases osteoporosis odds by approximately 17%, making age the dominant driver of the model. Aside from age, only corticosteroid medication use showed a statistically significant association (χ^2^ = 5.04; *p* = 0.025; OR = 1.36), although its effect size was much smaller. All other lifestyle, demographic, nutritional, and clinical variables—including gender, hormonal status, family history, race/ethnicity, body weight, calcium intake, vitamin D intake, smoking, alcohol consumption, physical activity, medical conditions, and prior fractures—showed non-significant effects (*p* > 0.05).

Findings from the combined likelihood ratio tests and logistic regression coefficients clearly indicate that age is the dominant predictor of osteoporosis, whereas most other demographic, lifestyle, and nutritional variables show minimal or no contribution to the model. Variables such as Gender, Family History, and Alcohol Consumption demonstrated extremely small χ^2^ values (0.0047–0.0395) and non-significant regression coefficients (*p* > 0.80), with odds ratios close to 1.0. These results suggest that their influence on osteoporosis risk within this dataset is negligible.

Based on this evidence, two screening models were developed:

1. Full Model (Comprehensive Model)

The full model incorporates all available predictors, including demographic, clinical, lifestyle, nutritional, and medical variables. This model serves as the baseline reference, capturing the maximum achievable predictive performance when the complete feature set is utilized.

2. Reduced Model (Simplified Screening Model)

The reduced model excludes Gender, Family History, and Alcohol Consumption, as these predictors demonstrated consistently non-significant effects, trivial effect sizes, and minimal contribution to the explanatory power of the model. By removing these low-impact features, the model achieves improved parsimony and interpretability, while maintaining performance nearly identical to the full model. This configuration is especially suitable for real-world or community-based screening, where data availability may be limited.

3. Two-Feature Model (Minimal Predictive Model)

To further identify the most essential predictors, a third model was developed using only Age and Medications (corticosteroid use)—the only two variables that showed statistically significant associations in both the omnibus likelihood ratio tests and logistic regression analyses. Age exhibited a dominant effect (OR ≈ 1.17 per year and *p* < 0.001), while corticosteroid use showed a modest but meaningful association (OR ≈ 1.36 and *p* = 0.025), consistent with established evidence regarding steroid-induced bone loss.

This model evaluates whether these two core predictors alone can sustain clinically acceptable screening performance. Given the negligible influence of most other variables—including demographic, lifestyle, and nutritional factors—the Two-Feature Model provides a highly parsimonious yet clinically coherent alternative. Preliminary comparisons indicate that overall discriminative capacity remains largely preserved, with only minor reductions, if any, in sensitivity or stability. This makes the model particularly advantageous for rapid screening, low-resource environments, telehealth workflows, or situations where comprehensive data collection is not feasible.

From Table 3, the comparison across the three model configurations—Full Model, Reduced Model, and the Two-Feature Model—demonstrated that screening performance remained highly stable despite progressive reductions in the feature set. The Full Model and Reduced Model showed nearly identical results, with accuracy values of 0.826 and 0.825, sensitivities of 0.781 in both cases, and specificities of 0.870 and 0.869, respectively. Their AUC values were also identical at 0.906, indicating that the removal of Gender, Family History, and Alcohol Consumption had no meaningful impact on discriminative performance.

The Two-Feature Model, which included only Age and Medications, exhibited performance that was similarly strong, achieving accuracy of 0.827, sensitivity of 0.783, specificity of 0.870, and an AUC of 0.904. Although the AUC was slightly lower than that of the full and reduced models, the difference was minimal and did not materially affect overall classification capability. Model fit indices showed expected patterns: while the Two-Feature Model had a slightly higher Deviance, its AIC and BIC values were lower than those of the Full Model, reflecting improved parsimony with only a marginal trade-off in fit.

Taken together, these results demonstrate that the core predictive capacity of the screening framework is preserved even when the model is reduced to only two variables, driven primarily by the strong effect of age. This supports the practical utility of simplified or minimal models, particularly for rapid osteoporosis screening in real-world, community-based, or resource-limited environments.

### 3.2. Feature Selection and Model Development Using Machine Learning

**Figure 3:**
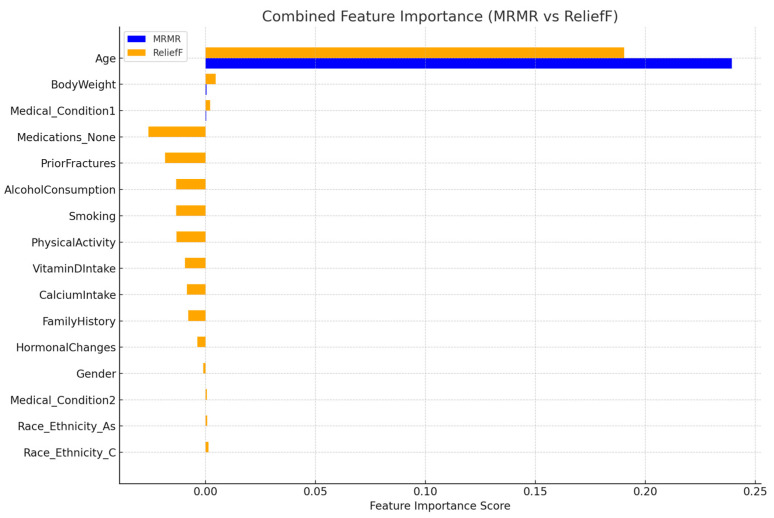
Combined Feature Importance Based on MRMR and ReliefF Algorithms.

From Figure 3, the bar plot compares feature importance scores derived from the MRMR and ReliefF feature selection methods across all predictors. Age consistently demonstrates the highest importance in both algorithms, indicating its dominant role in osteoporosis prediction. Other variables show minimal or near-zero contributions, reflecting low discriminative value within this dataset. Color coding distinguishes the two algorithms (MRMR in blue and ReliefF in orange), enabling direct comparison of ranking consistency.

To further evaluate the relative importance of predictors from a machine-learning perspective, feature importance was assessed using two complementary filter-based methods, namely Minimum Redundancy Maximum Relevance (MRMR) and ReliefF. The averaged importance scores derived from these methods were then used to rank all candidate features, as summarized in Table 4.

The combined analysis of feature importance using MRMR and ReliefF algorithms demonstrates a clear and consistent pattern in identifying the predictors most relevant to osteoporosis classification.

1. Age is the overwhelmingly dominant predictor

Age shows by far the highest importance score across both algorithms, with an average score more than 100 times greater than any other feature. This confirms that age contributes the most discriminative information in predicting osteoporosis, consistent with the logistic regression and model performance results.

2. Very small contributions from other variables

A few additional features—such as Medical_Condition1, Calcium Intake, Alcohol Consumption, and Physical Activity—received small positive scores, indicating minor influence. However, their importance values remain extremely low relative to age and are unlikely to meaningfully improve model performance.

3. Several variables show near-zero or negative importance

Many predictors, including Family History, Smoking, Hormonal Changes, Race/Ethnicity, and Body Weight, exhibit zero or negative ReliefF values. Negative scores indicate that the feature may add noise rather than improve classification, reinforcing their negligible predictive value in this dataset.

4. Strong agreement between MRMR and ReliefF

Both methods show a similar ranking pattern—clear dominance of age, small secondary contributions from clinical or nutritional factors, and weak influence from lifestyle and demographic variables. This cross-method consistency enhances confidence in the stability of feature relevance.

To evaluate the incremental contribution of key predictors and to identify the most parsimonious yet effective screening framework, five predictive model configurations were constructed based on increasing numbers of top-ranked features. The feature ranking was determined using a combination of MRMR scores and ReliefF scores with Age consistently emerging as the strongest predictor across all analyses.

Case 1: Full-Predictor Model (All features).
Case 2: Five-Predictor Model (5 Features):
  Age;
  Medications;
  Calcium Intake;
  Physical Activity;
  Alcohol Consumption (positive but small ReliefF score).
Case 3: Four-Predictor Model (4 Features):
  Age;
  Medications;
  Calcium Intake;
  Physical Activity (small positive ReliefF score).
Case 4: Three-Predictor Model (3 Features):
  Age;
  Medications;
  Calcium Intake (modest importance across ReliefF).
Case 5: Two-Predictor Model (2 Features):
  Age;
  Medications (corticosteroid use).
Case 6: Single-Predictor Model (1 Feature):
  Age.

Table 5 shows the comparison across the six feature selection cases reveals high stability in model performance, even as the number of predictors decreases from the full set down to a single-feature model. Across all configurations, the Tree, SVM, and KNN classifiers consistently achieved the highest validation accuracy—often above 91%, particularly in Cases 4–6. Notably, the single-predictor model (Case 6), which relied solely on Age, produced accuracy levels (≈91.42%) comparable to those of more complex models, further underscoring age as the dominant predictive factor. In addition to accuracy-based metrics, AUC values remained consistently strong across all models, typically ranging from 0.90 to 0.96, demonstrating excellent discriminative ability regardless of the number of features included. Importantly, even the most simplified models—Cases 5 and 6—achieved AUC values between 0.949 and 0.954, closely matching or exceeding those observed in the full-feature and intermediate-feature models. This stability in AUC reinforces the robustness of the minimal-predictor approach. Precision, recall, and F1-scores also remained remarkably stable across all cases, with top-performing models achieving values above 92%. Although training time increased substantially for Naïve Bayes and SVM in later cases, these computational differences did not affect predictive performance. The strongest and most efficient overall performers were Tree, SVM, and KNN, which maintained excellent classification quality across all feature configurations.

Case 5 evaluates a model using only two predictors—Age and Medications (corticosteroid use)—representing the smallest feature set that preserves statistically significant contributions from both variables. This model demonstrated excellent predictive performance, with the Tree, SVM, and KNN classifiers each achieving 91.42% accuracy, 92.68% precision, 91.42% recall, an F1-score of 91.36%, and AUC values around 0.954–0.949. These metrics are essentially identical to those obtained from more complex models (Cases 1–4), confirming that the inclusion of additional low-importance variables does not yield meaningful performance gains. In terms of efficiency, the Tree and KNN models trained substantially faster than Naïve Bayes and SVM, making them well suited for real-time or resource-constrained environments. The consistently high accuracy, precision, recall, F1-score, and AUC across classifiers indicate strong discriminative power and stable classification performance despite the simplicity of the model. Overall, Case 5 highlights the practical advantage of a highly parsimonious screening framework, demonstrating that a combination of just Age and corticosteroid medication use is sufficient to match the predictive accuracy and AUC of more complex feature sets. This finding reinforces the clinical and computational value of stability-driven feature selection and supports the deployment of ultra-minimal models in community hospitals, telemedicine screening platforms, and primary care settings.

Collectively, these results demonstrate that simplifying the model by reducing predictors does not compromise predictive accuracy or AUC. This supports the use of minimal or ultra-minimal feature sets—particularly models relying on Age alone or Age combined with Medications—as robust, efficient approaches for osteoporosis risk prediction, especially in resource-limited or community screening settings.

### 3.3. Consensus Feature Selection and Integrated Model Construction

From Table 6, a unified ranking integrating logistic regression effect estimates and machine learning importance scores consistently identified Age and Medications (corticosteroid use) as the dominant predictors across all analyses, while other variables showed substantially weaker combined contributions. This pattern suggests that minimal and ultra-minimal configurations primarily reflect established epidemiological risk factors rather than novel predictive signals.

Based on this unified assessment, additional multi-feature configurations were explored to evaluate whether secondary predictors with modest importance could provide incremental value. Variables such as Smoking, Prior Fractures, and selected Race/Ethnicity categories were sequentially incorporated according to their unified ranking (Estimate × Average Importance), ensuring a systematic and non-arbitrary expansion strategy. Although these factors were clearly weaker than the primary predictors, their inclusion allowed controlled assessment of potential additive effects and demographic interactions in more complex feature settings.

Case 7: Three-Predictor Model (3 Features):
  Age;
  Medications;
  Smoking.
Case 8: Four-Predictor Model (4 Features):
  Age;
  Medications;
  Smoking;
  PriorFractures.
Case 9: Five-Predictor Model (5 Features):
  Age;
  Medications;
  Smoking;
  PriorFractures;
  Race_Ethnicity_C.
Case 10: Four-Predictor Model (Rank 1–3 + Gender):
  Age;
  Medications;
  Smoking;
  Gender.
Case 11: Tree-Predictor Model (Rank 1–2 + Gender):
  Age;
  Medications;
  Gender.

Case 10 and Case 11 were specifically designed to evaluate the potential contribution of Gender, a well-established biological factor associated with osteoporosis risk, particularly in the context of postmenopausal bone loss. The inclusion of Gender in these configurations was motivated by extensive epidemiological evidence demonstrating pronounced sex-specific differences in osteoporosis prevalence and fracture risk, with postmenopausal women representing the most affected population group [47,48]. These cases were not intended to introduce a novel predictor, but rather to assess whether gender-related information provides incremental value within the proposed methodological framework.

From Table 7, across Cases 7–9, the inclusion of secondary predictors (e.g., Smoking, Prior Fractures, and Race/Ethnicity_C) maintained consistently high and stable model performance, with Case 7 in particular preserving strong predictive accuracy after adding Smoking. However, these additional features did not lead to substantial or systematic performance improvements compared with simpler configurations and, in some algorithms, resulted in increased computational cost. In contrast, Case 11 (Age + Medications + Gender) demonstrated a favorable balance between predictive performance and computational efficiency. While maintaining accuracy and AUC values comparable to other high-performing models, Case 11 required notably lower training time, making it an efficient minimal-feature configuration within the proposed methodological framework.

### 3.4. External Validation Using Retrospective Hospital Records

To assess the generalizability of the proposed models under distributional shift, external validation was performed using retrospective clinical records obtained from the University of Phayao Hospital. Four representative configurations—Case 5 (Age + Medications), Case 6 (Age only), Case 7 (Age + Medications + Smoking), and Case 11 (Age + Medications + Gender)—were selected for external testing across five machine learning algorithms. These cases were chosen to compare minimal and slightly expanded feature sets, as well as to evaluate the effect of incorporating clinically relevant secondary variables. Model robustness was assessed by examining performance on the external cohort, where population characteristics differed from those of the training dataset. The resulting accuracy, precision, recall, F1-score, and AUC values are summarized in Table 8.

As shown in Table 8, external validation resulted in a moderate decline in predictive performance compared with internal validation, which is expected under real-world conditions with differing population characteristics. Across the evaluated configurations (Cases 5, 6, 7, and 11), test-set accuracy generally ranged from approximately 73% to 77%, with precision and recall remaining relatively stable. While no single configuration consistently achieved the highest value across all performance metrics, Case 11 (Age + Medications + Gender) demonstrated a favorable overall balance between accuracy and discriminative ability. In particular, the Efficient Linear classifier in Case 11 showed competitive accuracy alongside relatively strong AUC values compared with other minimal and near-minimal models, making it a methodologically attractive option under external validation. These results indicate that minimal and near-minimal feature configurations retain acceptable predictive capability under distributional shift. Although performance attenuation is evident, the findings should be interpreted as preliminary evidence of robustness rather than definitive clinical generalizability. The combined evaluation of accuracy, AUC, and computational efficiency suggests that Efficient Linear models with carefully selected minimal features, such as Case 11, represent a promising balance within the proposed framework.

**Figure 4:**
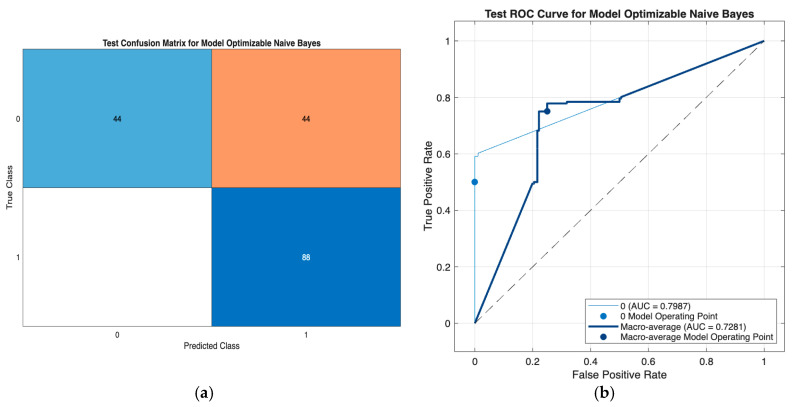

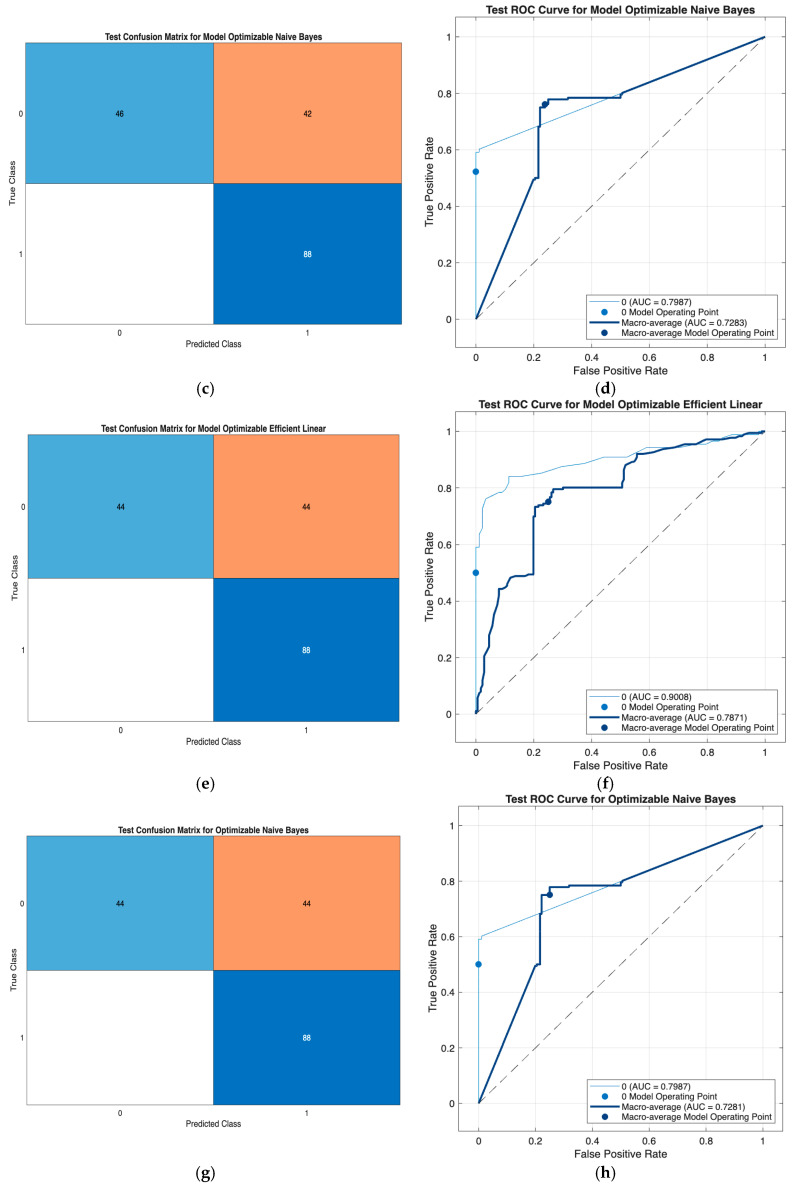

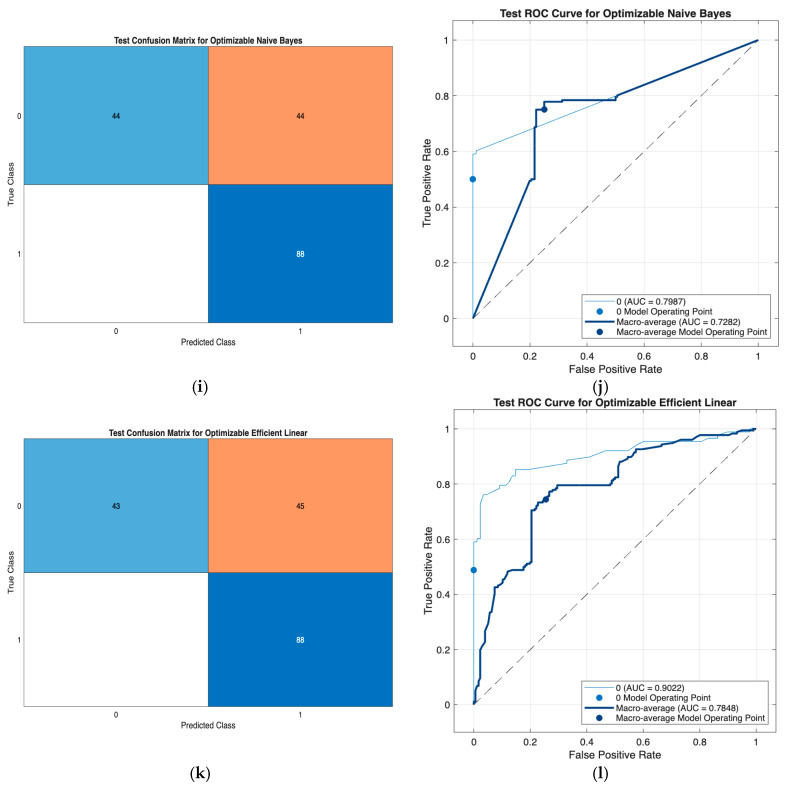
External test-set evaluation for Case 5–7, 11 models using Naïve Bayes and Efficient Linear classifiers. Panels (**a**,**c**,**e**,**g**,**i**,**k**) show the confusion matrices for the Optimizable Naïve Bayes (Case 5), Optimizable Naïve Bayes (Case 6), Optimizable Efficient Linear (Case 6), Optimizable Naïve Bayes (Case 7), Optimizable Naïve Bayes (Case 11), and Optimizable Efficient Linear (Case 11) models, respectively. Panels (**b**,**d**,**f**,**h**,**j**,**l**) show the ROC curve for the Optimizable Naïve Bayes (Case 5), Optimizable Naïve Bayes (Case 6), Optimizable Efficient Linear (Case 6), Optimizable Naïve Bayes (Case 7), Optimizable Naïve Bayes (Case 11), and Optimizable Efficient Linear (Case 11) models.

From Figure 4, external test-set evaluation demonstrated that both Naïve Bayes and Efficient Linear classifiers maintained stable and comparable performance across Cases 5–7 and 11. The confusion matrices indicate balanced classification behavior with consistent true-positive detection across models, while the ROC analyses show moderate to good discriminative ability under external validation.

Among the evaluated configurations, Case 11 (Age + Medications + Gender) exhibited a favorable balance between accuracy and AUC, particularly for the Efficient Linear classifier, which achieved competitive discrimination with a simple model structure. Overall, the results suggest that minimal and near-minimal feature models retain acceptable robustness under distributional shift, with Case 11 offering an efficient and methodologically attractive configuration for external evaluation.

All experimental configurations evaluated in this study are summarized in Table 9, encompassing models derived from statistical inference, machine learning feature importance, and their unified integration. This table provides a transparent overview of feature selection rationale and enables direct comparison across all cases.

## 4. Discussion

This study systematically examined the stability and predictive contribution of demographic, clinical, and lifestyle factors for osteoporosis risk prediction using an integrated framework combining statistical modeling, feature selection algorithms, and machine learning classifiers. Across all analytical approaches—including logistic regression, likelihood ratio testing, MRMR, ReliefF, and unified importance ranking—Age consistently emerged as the most dominant and stable predictor. Importantly, this dominance should be interpreted primarily as a dataset-driven effect, reflecting age imbalance and correlated feature structures within heterogeneous data sources, rather than as a novel or independent clinical discovery. In this context, age serves as an illustrative example of how a single variable can repeatedly surface across diverse machine learning pipelines due to underlying dataset characteristics, thereby influencing feature stability and apparent model sufficiency. While the observed prominence of age is consistent with well-established biological and epidemiological evidence regarding age-related decline in bone mineral density, the principal contribution of this study lies in demonstrating how such known risk factors behave within different feature selection frameworks under real-world data constraints.

Among secondary predictors, corticosteroid medication use was the only variable showing a statistically meaningful and biologically plausible association with osteoporosis, supporting its role as a clinically relevant complement to age. In contrast, other demographic and lifestyle variables—including gender, family history, race/ethnicity, nutritional intake, smoking, alcohol consumption, and physical activity—exhibited limited incremental discriminative value once age was accounted for, as reflected by weak effect sizes and consistently low feature-importance scores.

Machine learning experiments across multiple feature selection scenarios demonstrated that minimal and near-minimal models achieved predictive performance comparable to more complex multi-feature configurations. Notably, both the single-predictor model (Age only) and the two-predictor model (Age plus Medications) maintained strong internal performance. This observation should be interpreted primarily as a dataset-driven effect, reflecting age imbalance and correlated feature structures within the analyzed data, rather than as evidence of a novel or exclusive clinical role of age. In this context, the dominance of age illustrates how heterogeneous data distributions can lead to the consistent emergence of a single variable across machine learning pipelines, thereby influencing feature stability and apparent model sufficiency. These findings reinforce the methodological value of parsimony while highlighting the importance of cautious interpretation when dominant predictors arise from dataset characteristics rather than independent biological discovery.

External validation using retrospective clinical records further demonstrated that model performance decreased under distributional shift, as expected, yet remained acceptable. Across the evaluated configurations, Naïve Bayes and Efficient Linear classifiers showed the most stable external behavior. In particular, models incorporating Age, Medications, and Gender (Case 11) achieved a favorable balance between accuracy and discriminative ability, while maintaining lower computational cost, making them methodologically attractive for external evaluation under constrained data settings.

Importantly, these findings should be interpreted as methodological validation of stability-driven feature selection rather than evidence of newly identified clinical risk factors or readiness for clinical deployment. Simplified models should therefore be regarded as complementary analytical tools for exploratory or preliminary risk stratification, not as standalone screening or diagnostic instruments.

Future research should focus on larger, multi-center cohorts with greater demographic heterogeneity, alongside the integration of additional high-value data sources—such as imaging biomarkers and longitudinal fracture outcomes—to more rigorously assess generalizability, stability, and translational relevance.

## 5. Conclusions

This study demonstrates that stability-driven feature selection, integrated with statistical and machine learning analyses, enables the development of parsimonious and methodologically robust models for osteoporosis risk prediction. Across all analytical frameworks, age consistently emerged as the most reliable predictor, while corticosteroid use was the only additional variable providing meaningful and statistically significant predictive value. Other demographic, nutritional, and lifestyle factors contributed limited incremental information once age was accounted for. In addition, models incorporating gender alongside age and medication use—while still maintaining a near-minimal feature set—showed improved screening performances in selected external validation scenarios. This indicates that gender-related information can enhance discrimination without substantially increasing model complexity. Overall, minimal and near-minimal models, including age-only, age-plus-medication, and age–medication–gender configurations, achieved predictive performances comparable to more complex models. External validation confirmed that these streamlined models retain acceptable performances under population heterogeneity, although results should be interpreted as methodological evidence rather than justification for standalone clinical screening or diagnostic deployment. Future studies should extend validation to larger, multi-center cohorts and integrate longitudinal outcomes and imaging-derived biomarkers to further assess generalizability and clinical relevance.

## Figures and Tables

**Table 1:** Baseline Characteristics of Participants (N = 1958).

Characteristic	Non-Osteoporosis (*n* = 979)	Osteoporosis (*n* = 979)	Total
Age (years)	Mean = 24.3 ± 6.11 Median = 22	Mean = 53.9 ± 21.0 Median = 53	—
Gender			
Male	490 (50.1%)	502 (51.3%)	992 (50.7%)
Female	489 (49.9%)	477 (48.7%)	966 (49.3%)
Hormonal Changes			
Normal	25.4%	24.7%	50.1%
Postmenopausal	24.6%	25.3%	49.9%
Family History of Osteoporosis	Balanced (%)	Balanced (%)	—
Race/Ethnicity			
African American	17.2%	17.6%	34.8%
Asian	16.2%	16.0%	32.2%
Caucasian	16.6%	16.4%	33.0%
Body Weight Category			
Underweight	22.9%	24.7%	47.6%
Normal	27.1%	25.3%	52.4%
Calcium Intake			
Adequate	24.3%	24.5%	48.8%
Low	25.7%	25.5%	51.2%
Vitamin D Intake			
Sufficient	25.4%	26.3%	51.7%
Insufficient	24.6%	23.7%	48.3%
Physical Activity			
Active	26.6%	25.6%	52.2%
Sedentary	23.4%	24.4%	47.8%
Smoking Status			
Smoker	25.5%	24.7%	50.2%
Non-Smoker	24.5%	25.3%	49.8%
Alcohol Consumption			
Yes	24.7%	24.8%	49.5%
No	25.3%	25.2%	50.5%
Medical Conditions			
Hyperthyroidism	17.1%	17.5%	34.6%
Rheumatoid Arthritis	16.1%	16.2%	32.3%
None	16.8%	16.2%	33.0%
Medications			
Yes	24.0%	25.7%	49.7%
No	26.0%	24.3%	50.3%
Prior Fractures			
Yes	24.7%	25.5%	50.2%
No	25.3%	24.5%	49.8%

**Table 2:** Combined Summary of Predictors from Logistic Regression and Likelihood Ratio Tests.

Predictor	χ^2^ (LR Test)	*p* (LR)	Estimate	SE	Z	*p* (Model)	OR	95% CI (Lower–Upper)
Age	1329.604	<0.001	0.15794	0.00774	20.4029	<0.001	1.17109	1.15346–1.18900
Gender (Female–Male)	0.00473	0.945	0.00936	0.13609	0.0688	0.945	1.00940	0.77307–1.31798
Hormonal Changes (Postmenopausal–Normal)	0.17491	0.676	0.05686	0.13596	0.4182	0.676	1.05851	0.81090–1.38172
Family History (Yes–No)	0.02048	0.886	0.01946	0.13599	0.1431	0.886	1.01965	0.78108–1.33109
Race/Ethnicity (Asian–African American)	1.29513	0.523	0.19055	0.16771	1.1362	0.256	1.20992	0.87097–1.68078
Race/Ethnicity (Caucasian–African American)	—	—	0.10221	0.16689	0.6125	0.540	1.10762	0.79860–1.53621
Body Weight (Underweight–Normal)	0.28278	0.595	0.07245	0.13621	0.5319	0.595	1.07514	0.82323–1.40414
Calcium Intake (Adequate–Low)	0.04184	0.838	0.02782	0.13602	0.2046	0.838	1.02821	0.78759–1.34325
Vitamin D Intake (Insufficient–Sufficient)	0.44584	0.504	−0.09081	0.13605	−0.6675	0.504	0.91319	0.69945–1.19224
Physical Activity (Sedentary–Active)	0.32635	0.568	0.07784	0.13626	0.5712	0.568	1.08095	0.82760–1.41185
Smoking (Yes–No)	2.90776	0.088	-0.23205	0.13628	−1.7027	0.089	0.79291	0.60704–1.03568
Alcohol Consumption (Moderate–None)	0.03952	0.842	-0.02705	0.13609	−0.1988	0.842	0.97331	0.74543–1.27085
Medical Conditions (Hyperthyroidism–None)	1.36541	0.505	−0.02502	0.16450	−0.1521	0.879	0.97529	0.70650–1.34634
Medical Conditions (Rheumatoid Arthritis–None)	—	—	−0.18146	0.16728	−1.0848	0.278	0.83405	0.60009–1.15767
Medications (Corticosteroids–None)	5.04230	0.025	0.30505	0.13620	2.2397	0.025	1.35670	1.03884–1.77181
Prior Fractures (Yes–No)	2.31025	0.129	0.20731	0.13654	1.5182	0.129	1.23036	0.94147–1.60789

**Table 3:** Model performance for age as a continuous predictor (cut-off = 0.5).

Model	Accuracy	Sensitivity	Specificity	AUC	Deviance	AIC	BIC	McFadden’s R^2^	Tjur’s R^2^
Full model	0.826	0.781	0.870	0.906	1377	1411	1506	0.493	0.551
Reduced model	0.825	0.781	0.869	0.906	1377	1405	1483	0.493	0.551
Two-Feature Model	0.827	0.783	0.870	0.904	1386	1392	1409	0.489	0.547

**Table 4:** Ranked Feature Importance (Average of MRMR and ReliefF Scores).

Rank	Feature	MRMR	ReliefF	Average Importance
1	Age	0.2393	0.1903	0.2148
2	Medical_Condition1	0.0002	0.0045	0.00235
3	CalciumIntake	0.0000	0.0021	0.00105
4	AlcoholConsumption	0.0000	0.0013	0.00065
5	PhysicalActivity	0.0000	0.0007	0.00035
6	BodyWeight	0.0004	−0.0260	−0.0128
7	Gender	0.0000	0.0006	0.00030
8	Race_Ethnicity_C	0.0000	−0.0011	−0.00055
9	Medical_Condition2	0.0000	−0.0085	−0.00425
10	PriorFractures	0.0000	−0.0037	−0.00185
11	VitaminDIntake	0.0000	−0.0079	−0.00395
12	Race_Ethnicity_As	0.0000	−0.0094	−0.00470
13	FamilyHistory	0.0000	−0.0133	−0.00665
14	Smoking	0.0000	−0.0134	−0.00670
15	Medications_None	0.0000	−0.0134	−0.00670
16	HormonalChanges	0.0000	−0.0184	−0.00920

**Table 5:** Performance comparison of five machine learning classifiers across six feature selection scenarios (Case 1–Case 6).

	Model Type	Training Time (s)	Accuracy % (Validation)	Precision %	Recall %	F1 Score %	AUC
Case 1	Tree	44.73	90.60	91.87	90.60	90.53	0.9507
	Naive Bayes	201.56	86.06	87.99	86.06	85.88	0.9363
	SVM	440.84	85.60	87.08	85.60	85.45	0.9181
	Efficient Linear	238.61	82.94	83.57	82.94	82.86	0.9159
	KNN	267.99	85.96	86.46	85.96	85.91	0.9231
Case 2	Tree	18.01	91.06	92.26	91.06	91.00	0.9540
	Naive Bayes	62.56	86.36	88.28	86.36	86.19	0.9384
	SVM	497.25	87.69	87.88	87.69	87.68	0.8751
	Efficient Linear	36.89	83.30	83.87	83.30	83.23	0.9189
	KNN	33.94	86.41	87.57	86.41	86.31	0.8970
Case 3	Tree	15.38	91.06	92.29	91.06	91.00	0.9527
	Naive Bayes	62.67	86.11	88.07	86.11	85.93	0.9386
	SVM	269.38	89.89	90.60	89.89	89.84	0.9041
	Efficient Linear	57.45	83.09	83.57	83.09	83.03	0.9193
	KNN	62.81	90.19	91.27	90.19	90.13	0.9362
Case 4	Tree	14.72	91.16	92.49	91.16	91.09	0.9541
	Naive Bayes	49.32	86.16	88.06	86.16	85.98	0.9386
	SVM	141.70	91.16	92.34	91.16	91.10	0.9218
	Efficient Linear	41.39	82.99	83.54	82.99	82.92	0.9193
	KNN	28.91	91.16	92.34	91.16	91.10	0.9459
Case 5	Tree	620.45	91.42	92.68	91.42	91.36	0.9544
	Naive Bayes	1215.56	86.41	88.24	86.41	86.25	0.9388
	SVM	808.84	91.42	92.68	91.42	91.36	0.9253
	Efficient Linear	42.60	83.40	83.67	83.40	83.37	0.9115
	KNN	62.26	91.42	92.68	91.42	91.36	0.9499
Case 6	Tree	12.67	91.42	92.68	91.42	91.36	0.9548
	Naive Bayes	644.58	86.21	87.79	86.21	86.07	0.9383
	SVM	1234.26	91.42	92.68	91.42	91.36	0.9094
	Efficient Linear	42.05	85.24	86.55	85.24	85.11	0.9187
	KNN	49.87	91.42	92.68	91.42	91.36	0.9541

**Table 6:** Unified ranking of osteoporosis predictors based on combined statistical (logistic regression estimates) and machine learning feature importance (average MRMR + ReliefF) scores.

Rank	Feature	Estimate (abs)	Average Importance	Combined Score
1	Age	0.15794	0.2148	0.1864
2	Medications (Corticosteroids)	0.30505	0	0.1525
3	Smoking	0.23205	−0.00670	0.1127
4	PriorFractures	0.20731	−0.00185	0.1027
5	Race_Ethnicity_C	0.19055	−0.00055	0.095
6	Medical_Condition2	0.18146	−0.00425	0.0886
7	Race_Ethnicity_As	0.10221	−0.00470	0.0484
8	VitaminDIntake	0.09081	−0.00395	0.0434
9	PhysicalActivity	0.07784	0.00035	0.0391
10	BodyWeight	0.07245	−0.0128	0.0298
11	HormonalChanges	0.05686	−0.00920	0.0238
12	CalciumIntake	0.02782	0.00105	0.0144
13	AlcoholConsumption	0.02705	0.00065	0.0138
14	Medical_Condition1	0.02502	0.00235	0.0137
15	Medications_None	0.0305	−0.00670	0.0119
16	FamilyHistory	0.01946	−0.00665	0.0064
17	Gender	0.00936	0.0003	0.0048

**Table 7:** Performance of machine learning classifiers across three expanded feature selection scenarios (Case 7–11).

	Model Type	Training Time (s)	Accuracy % (Validation)	Precision %	Recall %	F1 Score %	AUC
Case 7	Tree	23.61	91.42	92.68	91.42	91.36	0.9542
	Naive Bayes	40.43	86.31	88.13	86.31	86.15	0.9388
	SVM	135.09	91.42	92.68	91.42	91.36	0.9293
	Efficient Linear	56.37	83.66	84.26	83.66	83.58	0.9182
	KNN	57.79	91.42	92.68	91.42	91.36	0.9447
Case 8	Tree	19.95	91.01	92.11	91.01	90.95	0.9515
	Naive Bayes	61.32	86.36	88.17	86.36	86.20	0.9389
	SVM	294.90	89.89	90.65	89.89	89.84	0.9023
	Efficient Linear	40.41	83.71	84.44	83.71	83.62	0.9194
	KNN	36.23	90.30	91.46	90.30	90.23	0.9338
Case 9	Tree	21.20	91.16	92.25	91.16	91.11	0.9521
	Naive Bayes	59.68	86.21	88.06	86.21	86.04	0.9387
	SVM	544.23	87.28	87.40	87.28	87.27	0.8747
	Efficient Linear	39.76	83.55	84.23	83.55	83.47	0.9184
	KNN	32.98	88.76	89.44	88.76	88.72	0.9204
Case 10	Tree	1.30	90.81	91.87	90.81	90.75	0.9214
	Naive Bayes	44.63	86.47	88.21	86.47	86.31	0.9199
	SVM	229.33	89.99	91.09	89.99	89.92	0.9133
	Efficient Linear	53.03	83.66	84.40	83.66	83.57	0.9137
	KNN	69.55	91.01	92.11	91.01	90.95	0.9132
Case 11	Tree	1.16	91.42	92.68	91.42	91.36	0.9238
	Naive Bayes	30.44	86.41	88.28	86.41	86.25	0.9200
	SVM	907.67	91.01	92.11	91.01	90.95	0.9107
	Efficient Linear	765.44	82.58	82.75	82.58	82.56	0.8940
	KNN	585.78	91.42	92.68	91.42	91.36	0.9192

**Table 8:** External validation results of minimal-feature models (Case 5 and Case 6) using retrospective hospital records.

Model Number	Model Type	Accuracy % (Validation)	Accuracy % (Test)	Precision % (Test)	Recall % (Test)	F1 Score % (Test)	AUC
Case 5	Tree	91.42	74.43	83.08	74.43	72.64	0.6818
	Naive Bayes	86.41	75.00	83.33	75.00	73.33	0.7281
	SVM	91.42	74.43	83.08	74.43	72.64	0.7627
	Efficient Linear	83.40	73.30	82.59	73.30	71.24	0.7670
	KNN	91.42	74.43	83.08	74.43	72.64	0.6818
Case 6	Tree	91.42	74.43	83.08	74.43	72.64	0.6818
	Naive Bayes	86.21	76.14	83.85	76.14	74.70	0.7282
	SVM	91.42	74.43	83.08	74.43	72.64	0.7306
	Efficient Linear	85.24	75.00	83.33	75.00	73.33	0.7870
	KNN	91.42	74.43	83.08	74.43	72.64	0.6818
Case 7	Tree	91.42	74.43	83.08	74.43	72.64	0.7443
	Naive Bayes	86.31	75.00	83.33	75.00	73.33	0.7987
	SVM	91.42	74.43	83.08	74.43	72.64	0.7457
	Efficient Linear	83.66	74.43	83.08	74.43	72.64	0.9017
	KNN	91.42	74.43	83.08	74.43	72.64	0.7443
Case 11	Tree	91.42	74.43	83.08	74.43	72.64	0.7443
	Naive Bayes	86.41	76.14	83.33	75.00	73.33	0.7987
	SVM	91.01	74.43	83.08	74.43	72.64	0.9107
	Efficient Linear	82.58	75.00	83.08	74.43	72.64	0.9022
	KNN	91.42	74.43	83.08	74.43	72.64	0.7443

**Table 9:** Summary of experimental cases based on statistical analysis, machine learning feature importance, and combined ranking strategies.

Case	Selection Basis	Model Description	Included Predictors	Methodological Purpose
Full Model	Statistical analysis (Table 2)	Comprehensive	All predictors	Baseline model capturing maximum explanatory power
Reduced Model	Statistical analysis (Table 2)	Simplified Screening Model	All except Gender, Family History, Alcohol	Improve parsimony and interpretability with minimal performance loss
Two-Feature Model	Statistical analysis (Table 2)	Minimal Predictive Model	Age, Medications (corticosteroids)	Minimal statistically supported screening model
Case 1	Feature importance (Table 4)	Full-Predictor Model	All predictors	Reference model for ML-based ranking
Case 2	Feature importance (Table 4)	Five-Predictor Model	Age, Medications, Calcium Intake, Physical Activity, Alcohol	Includes predictors with small positive ReliefF scores
Case 3	Feature importance (Table 4)	Four-Predictor Model	Age, Medications, Calcium Intake, Physical Activity	Removal of marginal predictors
Case 4	Feature importance (Table 4)	Three-Predictor Model	Age, Medications, Calcium Intake	Core ML-supported predictors
Case 5	Feature importance (Table 4)	Two-Predictor Model	Age, Medications	Highly parsimonious ML-driven model
Case 6	Feature importance (Table 4)	Single-Predictor Model	Age	Lower-bound benchmark
Case 7	Combined ranking (Table 6)	Three-Predictor Model	Age, Medications, Smoking	Unified statistical–ML ranking
Case 8	Combined ranking (Table 6)	Four-Predictor Model	Age, Medications, Smoking, Prior Fractures	Extended combined-ranking model
Case 9	Combined ranking (Table 6)	Five-Predictor Model	Age, Medications, Smoking, Prior Fractures, Race_Ethnicity_C	Extended combined-ranking model
Case 10	Combined ranking + Gender	Rank 1–3 + Gender	Age, Medications, Smoking, Gender	Explicit evaluation of Gender contribution
Case 11	Combined ranking + Gender	Rank 1–2 + Gender	Age, Medications, Gender	Isolated assessment of Gender in minimal setting

## Data Availability

The open-access dataset used in this study, Lifestyle Factors Influencing Osteoporosis, is publicly available on Kaggle and contains 1958 complete records comprising demographic, lifestyle, nutritional, hormonal, and medical variables relevant to osteoporosis risk. This dataset can be accessed at: https://www.kaggle.com/datasets/amitvkulkarni/lifestyle-factors-influencing-osteoporosis (accessed on 20 August 2025). The retrospective clinical dataset used for external validation was obtained from the University of Phayao Hospital and includes 176 patient records collected between 2018 and 2025. Due to ethical and institutional regulations, this hospital dataset is not publicly available, but may be accessed upon reasonable request to the corresponding author and with appropriate ethics approval.
